# Mechanistic study of the effect of Endothelin SNPs in microvascular angina – Protocol of the PRIZE Endothelin Sub-Study

**DOI:** 10.1016/j.ijcha.2022.100980

**Published:** 2022-02-25

**Authors:** George R. Abraham, Andrew J. Morrow, Joana Oliveira, Jonathan R. Weir-McCall, Emma E. Davenport, Colin Berry, Anthony P. Davenport, Stephen P. Hoole

**Affiliations:** a Royal Papworth Hospital NHS Foundation Trust., Papworth Road, Cambridge Biomedical Campus, Cambridge CB2 0AY, United Kingdom; b University of Cambridge, The Old Schools, Trinity Lane, Cambridge CB2 1TN, United Kingdom; c NHS Greater Glasgow and Clyde Health Board., Gartnavel Royal Hospital Campus, 1055 Great Western Road, Glasgow G12 0XH, United Kingdom; d British Heart Foundation Glasgow Cardiovascular Research Centre, University of Glasgow, BHF Glasgow Cardiovascular Research Centre (GCRC), 126 University Place, Glasgow G12 8TA, United Kingdom; e Wellcome Sanger Institute., Wellcome Genome Campus, Hinxton, Cambridge CB10 1SA, United Kingdom

**Keywords:** Microvascular angina, Endothelin-1, rs1878406, rs6842241, rs6841581, rs93449379

## Abstract

**Introduction:**

Microvascular angina is a common cause of ischemia with non-obstructive coronary arteries (INOCA) and limited therapeutic options are available to those affected. Endothelin-1 (ET-1) is a potent vasoconstrictor implicated in the pathophysiology of microvascular angina. A large randomised, double blinded, placebo controlled crossover trial, the PRecIsion medicine with ZibotEntan in microvascular angina (PRIZE) trial is currently underway, investigating an endothelin receptor antagonist – Zibotentan, as a new drug treatment for microvascular angina. The trial uses a 'precision medicine' approach by preferential selection of those with higher ET-1 expression conferred by the PHACTR1 minor G allele single nucleotide polymorphism (SNP). The incidence of this SNP occurs in approximately one third of the population therefore a considerable number of screened patients will be ineligible for randomisation and the treatment phase of the trial.

**Methods:**

In the PRIZE Endothelin (ET) Sub-Study, patients screened out of the PRIZE trial will be genotyped for other genetic variants in the ET-1 pathway. These will be correlated with phenotypic characteristics including exercise tolerance, angina severity and quantitative measures of microvascular function on cardiovascular MRI as well as mechanistic data on endothelin pathway signalling.

**Conclusions:**

The study will provide a comprehensive genotype and phenotype bio-resource identifying novel ET-1 genotypes to inform the potential wider use of endothelin receptor antagonists for this indication.

## Highlights

• Microvascular angina is a common cause of ischemia with non-obstructive coronary arteries (INOCA).
• Endothelin-1 (ET-1) is a potent vasoconstrictor implicated in the pathophysiology of microvascular angina.
• Zibotentan, an Endothelin Receptor Antagonist is being tested as a treatment for microvascular angina in the ‘PRIZE’ trial using a genetic ‘precision medicine’ approach.
• The PRIZE ET Sub-study will provide a comprehensive genotype and phenotype bio-resource for microvascular angina patients.

## 1 Introduction:

Ischaemic heart disease is the leading cause of premature disability and death worldwide, with stable angina pectoris the commonest symptomatic manifestation, affecting 112 million people [1], [2], [3]. Angina pectoris occurs due to a relative myocardial oxygen supply–demand mismatch, typically during physiological (e.g. exercise) or psychological stress. Historical teaching placed a causal link between obstructive epicardial coronary artery disease and angina. However, in patients undergoing coronary angiography with evidence of myocardial ischemia, the prevalence of non-obstructive coronary disease is reported in up to 70% with higher frequencies among women [2]. Cardiac ischemia in the absence of significant epicardial coronary stenosis is termed INOCA (Ischemia and No Obstructive Coronary Artery Disease). This group of patients experience long-term adverse outcomes including recurrent angina, cardiovascular death and myocardial infarction (MI) as well as lower quality of life and health anxiety causing repeated presentations to healthcare services [4], [5], [6], [7]. High quality trial data for drug therapy in this condition is lacking and standard anti-anginal therapy is unsatisfactory for many patients [2]. Based on previous studies of INOCA populations (typically excluding those whose ischemia is attributable to non-coronary cardiac or systemic diseases such as hypertrophic cardiomyopathy and valvular heart disease), it is estimated that in around two-thirds, coronary microvascular dysfunction (CMD) represents the underlying pathology [8], [9]. Microvascular angina is the name given for the clinical syndrome caused by CMD in these patients. The Coronary Vasomotion Disorders International Study (COVADIS) group have recently defined the diagnostic criteria for microvascular angina (Table 1) [10]. This definition of microvascular angina aligns with the term 'type 1 CMD' described initially by Camici and Crea [11]. The other principal INOCA endotype is epicardial vasospastic angina (VSA) which may co-exist with microvascular angina in some patients.

**Table 1:** COVADIS criteria for Microvascular Angina [10] used to identify patients eligible for PRIZE and the PRIZE ET Sub-Study. CAD indicates coronary artery disease; ECG, electrocardiogram; FFR, fractional flow reserve; Coronary CTA, CT coronary angiography; IMR, index of microcirculatory resistance; HMR, hyperemic microvascular resistance; SOB, shortness of breath; TIMI frame count refers to the scheme described by the Thrombolysis in Myocardial Infarction study group.

1. Symptoms of myocardial ischaemia	Effort angina or rest angina Angina equivalent e.g. SOB

2. Absence of obstructive CAD (>50% diameter reduction or FFR < 0.80) by	Coronary CTA Invasive coronary angiogram

3. Objective evidence of myocardial ischaemia	Ischemic ECG changes during an episode of chest pain Stress-induced chest pain and/or ischemic ECG changes in the presence or absence of transient/reversible abnormal myocardial perfusion and/or wall motion abnormality

4. Evidence of impaired coronary microvascular function	Impaired coronary flow reserve (cut-off values depending on methodology use between ≤ 2.0 and ≤ 2.5) Coronary microvascular spasm, defined as reproduction of symptoms, ischemic ECG shifts but no epicardial spasm during acetylcholine testing. Abnormal coronary microvascular resistance indices (e.g. IMR > 25, HMR ≥ 2.5 mm Hg·cm-1·s) Coronary slow flow phenomenon, defined as TIMI frame count > 25.
(4/4 = definite, 3/4 = probable diagnosis of microvascular angina)	

### 1.1 Detecting and defining CMD

Given patency of the epicardial coronary arteries and adequate aortic pressure, coronary blood flow is regulated by dynamic changes in the resistance of the microvascular pre-arterioles and arterioles which are too small to be visualised on conventional angiography (diameter ≤ 500 µm) [12]. Multiple mechanistic sub-types of CMD have been proposed encompassing deleterious structural remodelling of the microvasculature as well as functional vasomotor abnormalities; however, in all cases, the fundamental abnormality is an inability of the coronary microvasculature to adequately match coronary blood flow to increased demand, such as during exercise [13].

CMD can be detected by invasive coronary guidewire based measurements of coronary blood flow, for example: Coronary Flow Reserve (CFR), which is the ratio of coronary blood flow during pharmacologically induced hyperemia to resting flow [12], [13]. Cardiovascular Magnetic Resonance (CMR) imaging additionally permits the non-invasive detection and quantification of CMD through the use of myocardial stress perfusion imaging with gadolinium contrast [14], [15]. Hyperemic myocardial blood flow (MBF) is measured during administration of adenosine and compared with resting blood flow to determine the myocardial perfusion reserve (MPR). This ratio of hyperemic to resting blood flow is analogous to CFR, and has prognostic value in CMD [16]. CMR has high spatial resolution, allowing differences between the epicardial and subendocardial blood flow to be accurately detected and quantified [17].

### 1.2 Endothelin

Endothelin-1 (ET-1) is an endothelial peptide crucial for physiological regulation of vasomotor tone in coronary arteries [18], [19]. Increased circulating levels of ET-1 have been identified in patients with CMD [20], [21]. ET-1 is released from endothelial cells and causes potent and sustained vasoconstriction by binding to ET_A_ receptors expressed in vascular smooth muscle. These effects are counter-balanced in healthy individuals by the ET_B_ pathway, where vasodilators such as nitric oxide are released in response to ET-1 binding to endothelial ET_B_ receptors [22]. ET antagonists (across a spectrum of selectivity for ET_A_ versus ET_B_) have been investigated in a wide range of diseases. Clinical benefit has thus far, been best established in pulmonary arterial hypertension and three agents are currently licensed for this indication: bosentan, ambrisentan and macitentan [22].

Zibotentan (AstraZeneca, Cambridge, UK) is the most ET_A_ selective antagonist with high potency (pIC_50_ (negative logarithm of the half-maximal inhibitory concentration) for ET binding to ET_A_ receptors = 21 nM) [22] and is under investigation as a novel therapy for microvascular angina in the Medical Research Council's Precision Medicine with Zibotentan in Microvascular Angina (PRIZE) trial [23].

### 1.3 Genetic dysregulation of Endothelin-1 and microvascular angina

The PRIZE trial is notable amongst cardiovascular clinical trials for defining eligibility by genetic analysis thus invoking a ‘precision medicine’ pathway whereby therapy will be targeted at those most likely to benefit. The genetic variant tested during screening for the PRIZE trial is the G allele of the rs9349379 SNP (single nucleotide polymorphism) found in the third intron of the PHACTR1 gene on chromosome 6. This variant has been linked to cardiovascular risk via enhanced Endothelin (EDN1) gene expression and is relatively common, with an estimated population frequency of 36% [23], [24]. In a sub-study of the British Heart Foundation Coronary Microvascular Angina (CorMicA) trial, patients with the rs9349379-G allele had significantly increased circulating concentrations of ET-1 as well as a higher likelihood of CMD, more myocardial perfusion defects on CMR, and decreased exercise tolerance when compared to patients without [25].

**Fig. 1:**
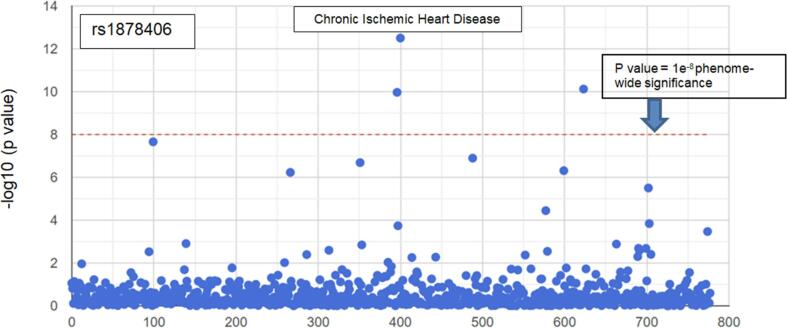
Phenome wide associations for rs1878406 with disease traits identified from the UK Biobank [30]. Traits (blue dots) above the red dotted line have a p value for association of < 1 × e^-8^. Chronic Ischemic Heart Disease (labelled) shows the strongest association. (For interpretation of the references to colour in this figure legend, the reader is referred to the web version of this article.)

**Fig. 2:**
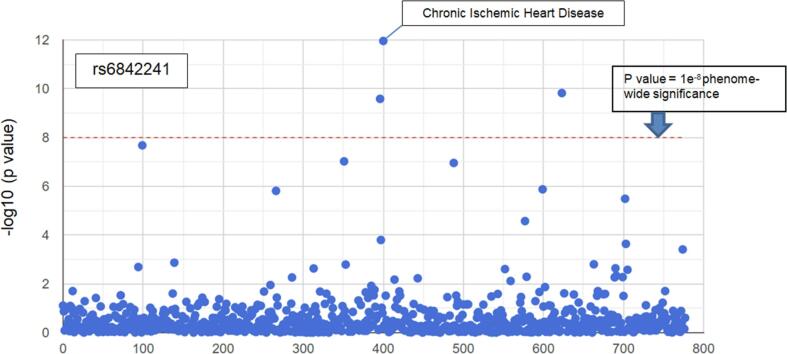
Phenome wide associations for rs6842241 with disease traits identified from the UK Biobank[30]. Traits (blue dots) above the red dotted line have a p value for association of < 1 × e^-8^. Chronic Ischemic Heart Disease (labelled) shows the strongest association. (For interpretation of the references to colour in this figure legend, the reader is referred to the web version of this article.)

The principal aim of the PRIZE ET Sub-study is to identify additional novel, hitherto unknown functional genetic variants associated with microvascular angina, other than the rs9349379-G allele described above. For this reason, the study will run in parallel with the main PRIZE trial by preferential enrolment of the patients who are not selected after genetic screening for the rs9349379-G allele. Patient samples obtained in this parallel study will be genotyped using the UK Biobank Axiom^TM^ DNA Microarray. Briefly, the process involves extraction of DNA from whole blood, which is then denatured and fragmented, flourescentally labelled, and added to the array – a laborotary plate embedded with hundreds of thousands of DNA ‘probes’ (specific oligonucleotide sequences complementary to the genomic SNPs of interest). In the presence of a complementary DNA sequence, binding will occur by hybridization and scanning of the array for specific patterns of fluorescence can be used to identify genomic SNPs present in the patient's DNA [26]. In our study, analysis will be focused in the first instance, on SNPs located close to the gene encoding the ET_A_ receptor (EDNRA) with highly significant GWAS associations for ischemic heart disease, namely rs1878406 (Fig. 1) and rs6842241 (Fig. 2).

The rs1878406 SNP is located 8.5 kb upstream from the EDNRA gene on chromosome 4. The major rs1878406-C allele occurs with a frequency of 85% in Caucasian populations and is associated with cardiovascular risk, whereas the T allele is protective [27], [28]. The rs6842241 SNP is located 1.25 kb upstream from the EDNRA gene within the regulatory region. The risk allele (G) of rs6842241 has a frequency of 83% and is highly correlated (r^2^ > 0.8) with both rs1878406 and another SNP, rs6841581 located further upstream [29], which has been been identified in functional analysis as a possible epigenetic regulator of EDNRA gene expression [29].

We hypothesise that in a microvascular angina population, these SNPs related to the ET_A_ receptor, will result in greater ET_A_ receptor activation, reduced myocardial perfusion and more angina. Additionally, we wish to investigate whether higher ET_A_ receptor expression is associated with more severe disease, even amongst those without elevated circulating ET-1 levels.

## 2 Methods and analysis

### 2.1 Patient selection:

**Fig. 3:**
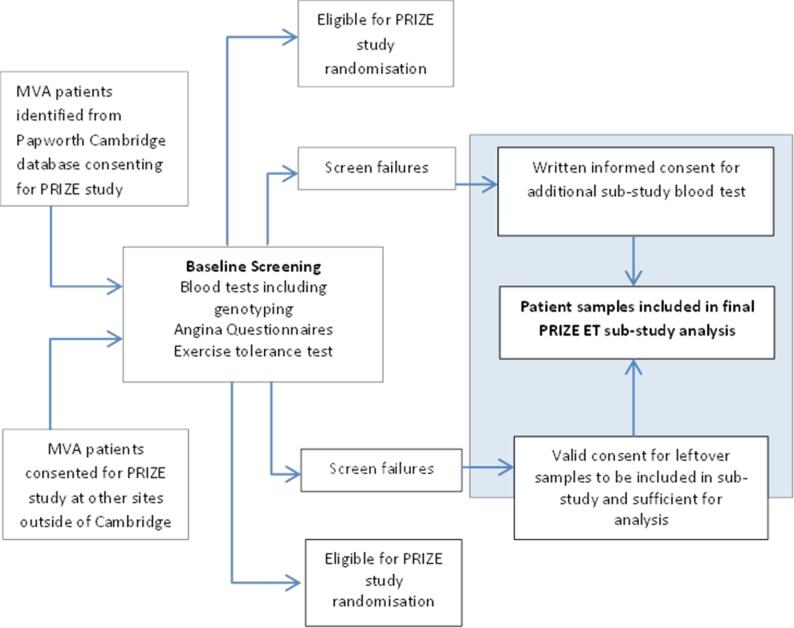
Flowchart for PRIZE Endothelin (ET) Sub-Study (blue box). MVA corresponds to microvascular angina. (For interpretation of the references to colour in this figure legend, the reader is referred to the web version of this article.)

The PRIZE trial will invite up to 356 participants from 8 UK sites for genetic testing and eligibility for progression into the randomisation phase. In Cambridge, approximately 100 patients will be enrolled into the PRIZE study. Once the results of genotype analysis and other baseline screening tests are available, it is expected there will be 66 screen failures and these patients will be approached to continue in the PRIZE Endothelin (ET) sub-study rather than the main PRIZE study (Fig. 3). This will include genetic screen failures as well as those found to have exclusion criteria during clinical assessment. We anticipate most of these patients (n = 60) will re-consent for the sub-study.

In Cambridge, patients will be initially identified from the Royal Papworth Hospital NHS Foundation Trust Microvascular Angina database as well as local participant identification centres and referring district general hospitals having met the definite or probable diagnostic criteria of microvascular angina as defined by the COVADIS group summarised in Table 1. Patients found to have the inclusion criteria for the PRIZE trial but subsequently excluded after genetic testing and baseline screening will be eligible for the PRIZE ET Sub-Study.

Participants may be screened out of the main PRIZE trial on the basis of their rs9349379 genetic sequencing blood test undertaken centrally in the NHS West of Scotland Centre for Genomic Medicine, Queen Elizabeth University Hospital, Glasgow. The patients and the researchers are blinded to the genotype. The result is then made available to the Clinical Trials Unit (Robertson Centre for Biostatistics, University of Glasgow). The PRIZE trial electronic case report form (eCRF) operates a 'genotype filter' which determines the participant's eligibility to continue in the main trial according to pre-determined, adaptively managed conditions with the final objective of achieving an overall 50% G allele frequency [23]. Participants who have been rejected by the genotype filter will be informed and then approached to participate in the PRIZE ET Sub-Study.

Participants may also have an exclusion criterion for the main PRIZE study (appendix A) during the first screening visit (i.e., before the results of the genetic blood test are available). For example, this may be because their total exercise time exceeds the maximum specified in the exclusion criteria. These participants will be offered the option of taking part in the PRIZE ET Sub-Study and the researchers will explain the rationale and study protocol. The participant will be allowed sufficient time to read the participant information sheet and ask any questions. If the patient provides informed consent, a venepuncture for the sub-study specific blood tests will be undertaken on the same visit.

### 2.2 Study procedures

Study blood handling will follow a standard operating procedure. Approximately 20 mL of venous blood will be withdrawn and collected. This will encompass a whole blood sample (4 mL) collected in PaxGene DNA tubes for DNA extraction and genotyping of ET_A_ SNPs, a second whole blood sample collected in PaxGene RNA tubes (2.5 mL) for quantification of ET_A_ mRNA and a 5 mL plasma sample for measurement of plasma endothelin related peptides. Blood will be centrifuged at 1500g for 20 mins at 4 °C. Plasma will be siphoned, aliquoted and stored at −80° ± 10°.

The PRIZE trial baseline clinical assessment includes patient interview, angina, quality of life and treatment satisfaction questionnaires, and Bruce protocol treadmill exercise testing (a full list of baseline assessments is provided in appendix B). These will be repeated at subsequent trial visits only if there have been significant changes in clinical status from initial screening otherwise results from these assessments will be used in the sub-study analyses.

SNP genotyping will be undertaken using the UK Biobank Axiom^TM^ Array. ET_A_ mRNA will be quantified in leucocytes and platelets within the samples using quantitative real-time polymerase chain reaction (qPCR). Big ET-1 and ET-1 will be measured by enzyme-linked immunosorbent (ELISA) immunoassay.

### 2.3 Additional participant analyses

If valid consent for the sub-study procedures is available for participants recruited to PRIZE from sites outside of Cambridge, they are found to be eligible, and surplus stored frozen samples are available, these will be similarly analysed.

### 2.4 MRI analysis for quantification of microvascular dysfunction

We will analyse enrolled patients' standard of care perfusion MRI prior to genetic analysis. Stress myocardial perfusion will be quantified globally, and as an endocardial to epicardial perfusion ratio. Where rest perfusion has been performed, MPR will be calculated.

## 3 Statistical consideration

For sample size calculation, we considered a difference of 1.0 mL·g^−1^·min^−1^ for stress MBF on CMR clinically significant with a pooled standard deviation of 0.56 based on previous published data [14]. Additionally, based on pilot data from our laboratory, we have assumed a difference in circulating plasma ET-1 between patient groups of 0.4 pg/ml with a pooled standard deviation of 0.36 to be clinically meaningful. Power calculations for an independent samples *t*-test demonstrate that a sample size of 60 (estimated to give 51 patients with the risk associated rs1878406-C allele) will have > 80% power to detect significant differences in both stress MBF and in plasma ET-1 between patient genotypes with a level of significance of 0.05 for an alpha error (SPSS, IBM Corp., USA).

Normality of continuous variable distributions will be assessed using the one sample Kolmogorov-Smirnov Test. Continuous variables will be summarised as mean ± standard deviation if normally distributed and median (Inter-Quartile Range) if non-normally distributed. Appropriate paired difference tests will be used to compare continuous variables according to the normality of the distribution. The primary outcome measure will be the difference in the circulating concentrations of ET-1 related peptides and ET_A_ mRNA between observed genotypes. Secondary outcome measures will be exploratory, including differences in disease burden measured by questionnaires, exercise tolerance tests and perfusion CMR between various ET_A_ SNP genotypes. Clinical trials unit and data management support and storage will be via the use of OpenClinica.

## 4 Ethics and dissemination plan

This study has been approved by the Leicester South Research Ethics Committee (REC: 20/EM/0279) and recorded in the NIHR Central Portfolio Management System (CPMS: 47557). The study is registered on clinicaltrials.gov (NCT04508998). Patients recruited into the PRIZE ET Sub-Study locally will provide written informed consent before participation. Where appropriate, and if patients give their consent, remaining blood samples will be kept in storage for other analyses in ethically approved research studies. Patients participating in the main PRIZE trial outside of Cambridge will be asked to give consent for their anonymised data and stored tissue samples to be used in alternative ethically approved research studies such as this sub-study. The results will be disseminated in academic forums and publication will be sought in peer-reviewed journals. Participating patients will be informed of the research outcomes in a lay summary of the final report.

## 5 Translational outlook and conclusion

Microvascular angina is a common underlying diagnosis amongst patients with INOCA. INOCA is increasingly recognised as a major contributor to the worldwide health burden of ischemic heart disease, yet currently, specific disease modifying therapy is lacking [2]. The ET-1 pathway is of interest as a therapeutic target and ET_A_ receptor antagonists are already in clinical use for pulmonary arterial hypertension. The investigational drug, Zibotentan, is the most ET_A_ selective antagonist and is under investigation in the PRIZE trial [23]. The PRIZE ET Sub-Study will follow on from the PRIZE trial by examining the effect of novel ET_A_ receptor associated SNPs on the ET-1 pathway. The sub-study will generate a genotype bio-resource identifying novel genotypes and phenotypes amongst microvascular angina patients that could be further validated in the UK Biobank as well as in the randomised PRIZE trial cohort. In the latter case, it could be used to identify other groups of patients likely to benefit from Zibotentan therapy using a 'precision medicine' approach. The bioresource will be uniquely valuable in several other ways. To our knowledge, this will be the largest genotyping study undertaken in a pre-specified microvascular angina cohort. The bioresource will also present the genetic data alongside downstream molecular data to specifically define the ET-1 pathway in these individuals. Finally, the bio-resource will also clinically characterise microvascular angina patients by CMR assessment of coronary microvascular dysfunction, treadmill exercise tolerance, angina symptoms and quality of life providing a comprehensive observational dataset for this condition.

## Declaration of Competing Interest

The authors declare the following financial interests/personal relationships which may be considered as potential competing interests: CB is employed by the University of Glasgow which holds consultancy and research agreements for his work with Abbott Vascular, AstraZeneca, Auxilius Pharma, Boehringer Ingelheim, Causeway Therapeutics, Coroventis, Genentech, GSK, HeartFlow, Menarini, Neovasc, Siemens Healthcare, and Valo Health. CB and APD are named on a patent submitted by the University of Glasgow on the use of Zibotentan for microvascular angina. University of Cambridge holds research grants for APD with the Medical Research Council and AstraZeneca. APD is a member of the Scientific Advisory Boards for ENB Therapeutics, Pharmazz Inc and Actelion.

## Figures and Tables

**Table:** 

Baseline patient assessments for screening
• Demographic details, past medical history and cardiovascular drug therapy
• Seattle Angina Questionnaire *(SAQ)*
• EuroQol questionnaire *(EQ-5D-5L)* • Brief Illness Perception Questionnaire *(Brief-IPQ)* • Patient health questionnaire *(PHQ4)*
• Treatment satisfaction questionnaire *(TSQM)*
• Bruce protocol exercise treadmill test (ETT)

## Appendix A: Inclusion and exclusion criteria for the main PRIZE trial.

Inclusion criteria:

1. Age > 18 years.
2. Microvascular angina (as per COVADIS criteria [10]).
3. Able to comply with study procedures.
4. Written informed consent.

Exclusion criteria:

1. Exercise tolerance > 540 s in men and > 430 s in women (i.e. actual exercise duration (s) achieved on the Bruce protocol commensurate with predicted or, lack of anginal symptoms and/or ST-segment depression (0.1 mV) limiting exercise.
2. Non-cardiovascular exercise-limiting problem e.g. morbid (or severe) obesity (BMI ≥ 40.0 kg/m2)
3. Genotype not available
4. Women who are pregnant, breast-feeding or of child-bearing potential (WoCBP) without a negative pregnancy test and who are unwilling to use highly effective contraception as defined below for the duration of the study treatment and 30 days after last dose of study drug.
5. Men who are sexually active with a WoCBP who are unwilling to use condoms or other highly effective methods of contraception for the duration of study treatment and for 14 weeks after last dose of study drug.
6. Heart failure (New York Heart Association Grade ≥ II i.e. mild symptoms and slight limitation during ordinary activity)
7. Recent (<6 months) myocardial infarction
8. A history of epilepsy, other CNS adverse events, neurologic symptoms or signs consistent with spinal cord compression or CNS metastases.
9. Moderate or more severe renal impairment (GFR < 45 mL/min)
10. Liver disease with a Child-Pugh score of A (5–6 points) or higher
11. Participation in another intervention study involving a drug within the past 90 days or 5 half-lives whichever is longer (co-enrolment in observational studies is permitted).

## Appendix B

Baseline assessments made as part of the screening visit in the main PRIZE trial.
